# Transcriptome Profiling and Weighted Gene Correlation Network Analysis Reveal Hub Genes and Pathways Involved in the Response to Polyethylene-Glycol-Induced Drought Stress of Two *Citrus* Rootstocks

**DOI:** 10.3390/biology13080595

**Published:** 2024-08-07

**Authors:** Emanuele Scialò, Angelo Sicilia, Alberto Continella, Alessandra Gentile, Angela Roberta Lo Piero

**Affiliations:** Department of Agriculture, Food and Environment, University of Catania, 951213 Catania, Italy; emanuele.scialo@phd.unict.it (E.S.); angelo.sicilia@unict.it (A.S.); alberto.continella@unict.it (A.C.); gentilea@unict.it (A.G.)

**Keywords:** drought, citrus, rootstock, PEG, RNA-Seq, WGCNA

## Abstract

**Simple Summary:**

Climate change poses a significant threat to agriculture, primarily through the increased frequency and severity of droughts. These extreme weather conditions reduce water availability, impacting crop yields and biomass production. Drought stress is particularly challenging for regions already prone to water scarcity, compromising food security. Addressing these issues requires innovative strategies and a thorough understanding of plant responses to water deficit conditions. This study aims to unravel the molecular mechanisms underlying drought tolerance in Citrus, crucial for developing adaptive strategies. The findings show that Bitters (C22) exhibits greater drought tolerance than Carrizo Citrange and identify key genes involved in responding to water scarcity. In conclusion, this research enhances our understanding of how citrus plants respond to water stress and identifies promising candidate genes. These insights will guide future research aimed at developing drought-tolerant plants.

**Abstract:**

Agriculture faces the dual challenge of increasing food production and safeguarding the environment. Climate change exacerbates this challenge, reducing crop yield and biomass due to drought stress, especially in semi-arid regions where Citrus plants are cultivated. Understanding the molecular mechanisms underlying drought tolerance in Citrus is crucial for developing adaptive strategies. Plants of two citrus rootstocks, Carrizo Citrange and Bitters (C22), were grown in aerated half-strength Hoagland’s nutrient solution. Post-acclimation, the plants were exposed to a solution containing 0% (control) or 15% PEG-8000 for 10 days. Leaf malonyl dialdehyde (MDA) and hydrogen peroxide (H_2_O_2_) content were measured to assess the reached oxidative stress level. Total RNA was extracted, sequenced, and de novo-assembled. Weighted Gene Correlation Network Analysis (WGCNA) was conducted to examine the relationship between gene expression patterns and the levels of MDA and H_2_O_2_ used as oxidative stress indicators. Plant visual inspection and MDA and H_2_O_2_ contents clearly indicate that Bitters is more tolerant than Carrizo towards PEG-induced drought stress. RNA-Seq analysis revealed a significantly higher number of differentially expressed genes (DEGs) in Carrizo (6092) than in Bitters (320), with most being associated with drought sensing, ROS scavenging, osmolyte biosynthesis, and cell wall metabolism. Moreover, the WGCNA identified transcription factors significantly correlated with MDA and H_2_O_2_ levels, thus providing insights into drought-coping strategies and offering candidate genes for enhancing citrus drought tolerance.

## 1. Introduction

With the global population projected to reach 9 billion by mid-century, the agricultural sector faces the challenge of increasing food production while safeguarding the environment and ecosystem functionality. This task is compounded by ongoing climate change, adversely impacting plant growth and leading to a considerable reduction in crop yield and biomass [1]. Due to limited rainfall and insufficient water availability, drought stress represents one of the most deleterious environmental factors contributing to substantial and unpredictable losses in global agricultural production [2,3]. This constraint is expected to escalate in frequency, intensity, and geographic range, and might represent an especially challenging circumstance for Citrus trees, widely grown in semi-arid regions such as southern Italy and Spain. These areas are well known for the occurrence of dry and hot summers often characterised by an elevated evaporative demand. Indeed, given their high water consumption, the growth, yield, and fruit quality of Citrus are reported to be significantly constrained by limited water resources [4,5,6]. Research on plant–water relations indicates that plants have evolved two main mechanisms of drought resistance: stress avoidance and tolerance. Stress avoidance involves the plant’s capacity to maintain a suitable water status in its tissues during progressive soil water deficit and aims to balance water uptake and water loss. This response relies on various strategies, such as stomatal closure, reduced leaf area and growth, deep rooting, accelerating leaf senescence, and increased water use efficiency [7]. Furthermore, photosynthesis declines due to the CO_2_ scarcity induced by the closure of stomata [8]. Under such conditions, excessive light triggers photo-oxidation, increasing the accumulation of reactive oxygen species (ROS) (hydrogen peroxide, singlet oxygen, and superoxide radicle) [9]. In particular, excessive levels of H_2_O_2_ might lead to the accumulation of MDA, an indicator of membrane lipid peroxidation. On the other hand, stress tolerance refers to the plant’s ability to partially dehydrate but remain viable and restart growth when rainfall resumes. Primary strategies contributing to drought tolerance include changes in tissue elasticity, osmotic adjustment, and efficient antioxidant capacity [10]. Experimental evidence indicates that drought tolerance in *Citrus* spp., related genera (e.g., *Poncirus*), and their hybrids (e.g., citranges and citrumelos) primarily relies on avoidance mechanisms. Additionally, some evidence suggests that citrus plants may also employ tolerance mechanisms to drought, including osmotic [11,12,13] and cell wall elasticity [14] adjustments. A common approach to enhance drought tolerance involves the employment of rootstocks known for their abiotic stress tolerance, a strategy adopted across various plant species [15,16,17,18], *Citrus* spp. included. A proper rootstock (a) may reduce the adverse effects of environmental stresses on the scion, increasing its resistance to drought conditions; (b) may enable plant long-term survival and fruit quality by improving the efficiency of natural (water and soil) resource utilisation; and (c) may decrease the use of chemical inputs [19,20]. Until the early 2000s, most of the Italian citriculture was almost exclusively based on the use of sour orange as a rootstock, which conferred high quality to the scion cultivars. Unfortunately, sour orange sensitivity to citrus tristeza virus (CTV) [21] has led to new plantings mostly using Troyer and Carrizo Citranges (*Citrus sinensis* (L.) Osb. × *Poncirus trifoliata*), and Swingle citrumelo (*Citrus paradisi* Macf. × *P. trifoliata*) [22]. However, these rootstocks are known to be salt-sensitive [23] and to have low tolerance to high-pH calcareous soils, which are typical of many growing areas of southern Italy. Recently, the University of California Riverside (UCR) released Sunki mandarin (*Citrus sunki* Hort. ex Tan.) × trifoliate orange (*P. trifoliata*) hybrid rootstock, namely, Bitters (C22), showing good performance in many trials in terms of yield efficiency and high tolerance to calcareous soils [22]. Furthermore, Bitters has emerged as a promising suitable rootstock candidate, displaying higher tolerance to PEG-induced drought stress than Carrizo Citrange, the latter also exhibiting low efficiency in soil water utilisation [15,24]. Previous efforts in the drought tolerance screening of citrus species were restricted to in vivo and in vitro testing [13,25]. Alternatively, hydroponic platforms have been increasingly employed for rapid and effective drought screening of agronomic crops. However, relatively few studies have been conducted on fruit crops using hydroponic systems. Drought stress can be artificially induced through various strategies, such as water restriction [26], abscisic acid (ABA) treatment, and the application of polyethylene glycol (PEG) [27]. Among these methods, the use of PEG, a non-ionic water-soluble polymer, is widely favoured as it is not expected to penetrate plant cells [28]. In particular, Ziogas and co-workers analysed the proteome reprogramming resulting from the imposition of drought stress conditions in citrus plants using 15% (*w*/*v*) PEG-6000 application for 21 days to replicate drought stress conditions [29]. Mass spectrometry analysis enabled the identification of 42 differentially expressed proteins in PEG-treated plants and the majority of those proteins were downregulated, especially proteins involved in the photosynthetic process [29]. However, the global transcriptomic response of citrus to PEG-induced drought stress still remains unexplored. The widespread use of high-performance RNA sequencing platforms to dissect the regulatory mechanisms of plants under specific conditions has become a cornerstone for enhancing our comprehension of plant responses to adverse conditions such as drought stress, contributing to the isolation of genes of interest, creation of functional markers, and quantification of gene expression [30,31,32,33], especially when it is coupled with Weighted Gene Correlation Network Analysis (WGCNA) [34]. WGCNA is a systems biology method that groups genes exhibiting similar expression patterns into modules based on both the correlation among gene expressions acquired via NGS and the interconnectedness of biological processes in plants. As a result, RNA-Seq analysis followed by WGCNA has been extensively employed to explore the biological connections between co-expression networks and plant traits. It also aids in the identification of key genes strongly linked to traits, which can serve as valuable biomarkers [35,36,37,38]. In this work, to deepen the knowledge and comprehension of the molecular responses to PEG-induced drought stress in sweet orange, the transcriptomes of two citrus rootstocks of different sensitivity to drought, namely, Carrizo Citrange and C22, were analysed using RNA-Seq analysis and the de novo RNA assembly approach. Notably, this study represents the first transcriptome analysis of both Carrizo Citrange and C22 rootstocks under PEG-induced drought stress, providing novel insights into the diverse strategies adopted by different *citrus* genotypes to cope with water scarcity. Furthermore, the relationship between the transcriptomic outcome and the amount of H_2_O_2_ and MDA [39], which can be accurately quantified and can be used as stress indicators, was revealed by WGCNA. As far as we know, this is the first time that transcriptomic data are correlated with stress indicator metabolites in citrus, leading to the discovery of hub genes that might function as markers of water stress.

## 2. Materials and Methods

### 2.1. Plant Growth and Experimental Design

One-year-old plants of two citrus rootstock genotypes (Carrizo Citrange [*C. sinensis* (L.) Osb. cv. *Washington navel* × *P. trifoliata* (L.) Raf.] and Bitters (C22) [*C. sunki* × *P. trifoliata*]) were grown hydroponically in aerated half-strength Hoagland’s nutrient solution under a 16 h photoperiod (250 μmole m^−2^ s^−1^) and a 24 ± 1 °C temperature. After seven days of acclimation, the plants of both rootstock genotypes were divided into two groups and transferred to half-strength Hoagland’s nutrient solution containing 0 (control, CK) or 15% PEG-8000 (PEG-treated, PEG) for 10 days to impose the drought stress condition. Each treatment (CK or PEG) was independently run in triplicate, and each replicate consisted of three individual plants. After ten days of PEG treatment, the young leaves of each replicate were randomly sampled, immediately frozen in liquid nitrogen, and stored at −80 °C for further analyses.

### 2.2. Measurement of MDA and H_2_O_2_ Content

The measurement of MDA content was carried out on 70 mg of leaf tissue according to the method described in López-Hidalgo et al. [40]. H_2_O_2_ determination was carried out on 500 mg of leaf tissue according to the method described in Velikova et al. [41].

### 2.3. Total RNA Extraction and cDNA Synthesis

The total RNA from young leaves was extracted using TRIzol^TM^ reagent (ThermoFisher Scientific, Waltham, MA, USA). RNA degradation and contamination were monitored on 1% agarose gels. RNA purity and concentration were checked using the NanoDrop spectrophotometer (ThermoFisher Scientific). Before sequencing, sample RNA integrity (RIN) was assessed using the Agilent Bioanalyzer 2100 system (Agilent Technologies, Santa Clara, CA, USA).

### 2.4. Library Preparation and Sequencing

After the QC procedures, sequencing libraries were generated using NEBNext^®^ Ultra™ RNA Library Prep Kit for Illumina^®^ (New England Biolabs, Ipswich, MA, USA) following the manufacturer’s recommendations. Briefly, mRNA was enriched using poly-T oligo-attached magnetic beads. Fragmentation was carried out using divalent cations under elevated temperature in NEBNext First Strand Synthesis Reaction Buffer (5×), followed by cDNA synthesis using random hexamers and M-MuLV Reverse Transcriptase (RNase H-). After first-strand synthesis, a custom second-strand synthesis buffer (Illumina) was added containing dNTPs, RNase H, and *Escherichia coli* polymerase I to generate the second strand by nick-translation. After the adenylation of 3’ ends of DNA fragments, NEBNext Adaptors with a hairpin loop structure were ligated to prepare for hybridisation. To select cDNA fragments preferentially 150~200 bp in length, the library fragments were purified with the AMPure XP system (Beckman Coulter, Beverly, MA, USA). Then, 3 µL USER Enzyme by NEB was used with size-selected, adaptor-ligated cDNA at 37 °C for 15 min followed by 5 min at 95 °C before PCR. Successively, PCR was performed with Phusion High-Fidelity DNA polymerase, Universal PCR primers, and Index (X) Primer. The library concentration was first quantified using a Qubit 2.0 fluorometer (Life Technologies, Carlsbad, CA, USA), and then diluted to 1 ng/µL before checking the insert size on an Agilent Bioanalyzer 2100 system (Agilent Technologies, Santa Clara, CA, USA). Cluster generation and sequencing were performed by Novogene Bioinformatics Technology Co., Ltd. (Beijing, China). After cluster generation, the libraries were sequenced on the Illumina HiSeq2000 platform to generate paired-end reads. Raw data (raw reads) of fastq format were first processed through in-house Perl scripts. In this step, clean data were obtained by removing reads containing adapters, reads containing poly-N, and low-quality reads. Then, the Q20, Q30, GC-content, and sequence duplication level of the clean data were calculated. All downstream analyses were based on clean, high-quality data.

### 2.5. De Novo Transcriptome Assembling and Gene Functional Annotation

De novo transcriptome assembly was accomplished using Trinity (2.6.6 version) with minKmerCov = 3, min_glue = 4. Then, Hierarchical Clustering was performed by Corset (1.09 version) to remove redundancy (parameter −f true, Default, −m 10) and the longest transcript of each cluster was selected as unigenes. Gene function was annotated based on the following databases (and software): National Center for Biotechnology Information (NCBI) non-redundant protein sequences (Nr) and NCBI nonredundant nucleotide sequences (Nt) (NCBI blast, version 2.9.0, e-value = 1e-5), Protein family (Pfam) (hmmscan, version HMMER 3.1, parameter e-value = 0.01), Clusters of Orthologous Groups of proteins (KOG/COG) and Swiss-Prot (Diamond, version 0.8.22, parameter e-value = 1e-5), Kyoto Encyclopedia of Genes and Genomes (KEGG) (Diamond, KAAS, version 0.8.22, parameter e-value = 1e-5), and Gene Ontology (GO) (blast2go, version b2g4pipe_v2.5, parameter e-value = 1e-6).

### 2.6. Quantification of Gene Expression and Differential Expression Analysis

The gene expression levels were estimated by RSEM (1.2.28 version) with bowtie2 mismatch 0 parameters to map the Corset-filtered transcriptome. For each sample, clean data were mapped back onto the assembled transcriptome and read counts for each gene were then obtained. Differential expression analysis between the control (CK) and treated (PEG) samples was performed using the DESeq2 R package (1.26.0 version). The resulting *p*-values were adjusted using the Benjamini and Hochberg’s approach for controlling the false discovery rate [42]. Genes retrieved by DESeq2 with an adjusted *p*-value < 0.05 were assigned as differentially expressed. A log_2_ Fold Change threshold of ±1 was adopted. The GO enrichment analysis of the differentially expressed genes (DEGs) was implemented by the GOseq and topGO R packages (1.32.0 and 2.32.0 versions, corrected *p*-value < 0.05) Wallenius non-central hyper-geometric distribution. Furthermore, all of the unigenes were submitted to the KEGG pathway database for the systematic analysis of gene functions. KOBAS software (v3.0 version, corrected *p*-value < 0.05) was used to test the statistical enrichment of the differentially expressed genes in the KEGG pathways [43].

### 2.7. Real-Time Validation of Selected DEG Candidates Using qRT-PCR

Total leaf RNA (2 µg) was reverse-transcribed using the SuperScript™ Vilo™ cDNA synthesis kit by ThermoFisher Scientific (Warrington WA1 4SR, UK). Real-time qRT-PCR was performed with PowerUp SYBR Green Master mix (ThermoFisher Scientific) and carried out in the Rotor-Gene Q (QIAGEN^®^, Venlo, The Netherlands). The quantification of the relative expression of a total of nine DEGs was performed in triplicate and the fold changes were calculated by the 2^−ΔΔCT^ method. Primers were designed using the Primer3web PCR primer design tool (version 4.1.0) and obtained by Eurofins genomics. The *Actin* housekeeping gene was used as an endogenous reference. The ΔΔCT was calculated by subtracting the ΔCT of the control sample from the ΔCT of the PEG-treated sample. The selected DEGs and their corresponding primer sequences are provided in Table S1.

### 2.8. Weighted Gene Correlation Network Analysis

Co-expression analysis was performed using the WGCNA package in R [35] to identify gene clusters with highly correlated expression profiles (hub genes). In detail, an adjacency matrix was created using FPKM values of the unigenes obtained by RNA-Seq, filtered considering a threshold of FPKM > 1 in at least one sample. The pickSoftThreshold function was used to choose the proper soft-thresholding power [35]. In particular, for each analysis, the lowest power for which the scale-free topology fit index reaches 0.90 was used. The specific WGCNA analysis parameters in this study were set as follows: soft powers β = 14, WGCNA “mergeCutHeight” = 0.25. The adjacency matrix was transformed into a topological overlap matrix (TOM) as well as the corresponding dissimilarity (1-TOM). Afterwards, a hierarchical clustering dendrogram of the 1-TOM matrix was constructed to classify similar genes’ expression into different gene co-expression modules. To achieve the high reliability of the results, the minimum number of genes was set to 30. The relationships between each module and the MDA and H_2_O_2_ levels were estimated by calculating Pearson’s correlation using the module eigengene values. Therefore, modules with high correlation coefficients and a correlation padj ≤ 0.05 were selected for subsequent analysis. Gene Significance (GS) and Module Membership (MM) were calculated and a threshold of 0.65 was applied. Modules with correlation significance exceeding 0.7 were selected for further analysis.

### 2.9. Statistical Analysis

The statistical analyses were performed using R Studio (R version 2023.9.1.494) [44]. The significance of differences for MDA and H_2_O_2_ measurements was tested by ANOVA (*p*-value < 0.05).

## 3. Results

### 3.1. Plant Phenotype and Quantification of MDA and H_2_O_2_ Content

**Figure 1:**
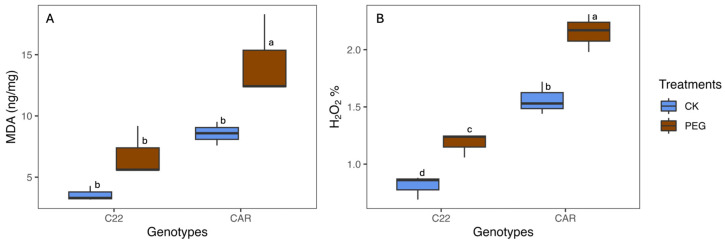
Malondialdehyde (MDA) (**A**) and hydrogen peroxide (H_2_O_2_) (**B**) content in treated (PEG) and control (CK) plants of two citrus rootstock genotypes after 10 days of PEG treatment. Each point represents the mean value of three replicates. Different letters indicate significantly different values (ANOVA, *p* < 0.05); CAR, Carrizo Citrange; C22, Bitters.

After 10 days of PEG treatment, the visual inspection of the plant phenotype revealed that Carrizo plants showed evident signs of water stress, including wilting and curled yellow leaves. In contrast, the Bitters plants exhibited milder symptoms, with their leaves retaining a greener hue and displaying less pronounced curling (Figure S1). To assess the level of oxidative stress, MDA and H_2_O_2_ content were measured as described in the Materials and Methods section. With regard to the MDA content, under drought stress conditions (PEG), Carrizo plants accumulated higher MDA levels than the control plants, whereas no significant differences were observed between the PEG-treated and CK plants in Bitters (Figure 1A). Moreover, the PEG-treated plants contained a higher amount of H_2_O_2_ content than the CK plants in both genotypes, reaching the highest level in PEG-treated Carrizo Citrange plants (Figure 1B).

### 3.2. Transcript Assembly and Annotation

Raw reads were filtered to remove reads containing adapters or reads of low quality so that the downstream analyses were based on a total of 387 million clean reads with an average of ~32 million reads per sample, with the average percentage of Q30 and GC being 91.1% and 44%, respectively (Table 1). The de novo assembly of clean reads resulted in 190,539 transcripts and 55,679 unigenes with N50 lengths of 2544 and 2384, respectively (Table 1), indicating that good contiguity of the transcriptome had been achieved. To evaluate the assembly consistency, the filtered unique reads were mapped back to the final assembled leaf transcriptome and the average read mapping rate, using the alignment software Bowtie2, was 77.22% (Table 1). Both transcript and unigene length distributions are reported in Figure S2. These data showed that the throughput and sequencing quality were high, allowing us to perform further analyses.

To achieve comprehensive gene functional annotation, all assembled unigenes were blasted against public databases, including the National Center for Biotechnology Information (NCBI), Protein family (Pfam), Clusters of Orthologous Groups of proteins (KOG/COG), Swiss-Prot, Ortholog database (KO) and Gene Ontology (GO) (Table 2). A total of 43,905 unigenes were annotated in at least one searched database, accounting for 78.85% of the obtained total unigenes. Among them, 34,859 (62.6%) and 36,131 (64.89%) assembled unigenes showed identity with sequences in the Nr and Nt databases, respectively. The percentage of assembled unigenes homologous to sequences in KO, Swiss-Prot, Pfam, GO, and KOG databases were 23.67, 47.77, 44.58, 44.58, and 16.05%, respectively (Table 2).

### 3.3. Identification of Differentially Expressed Genes (DEGs)

**Figure 2:**
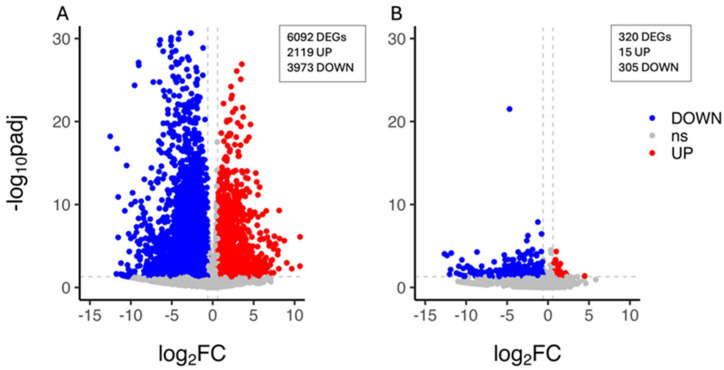
Volcano plot showing the differentially expressed genes (DEGs) in the CAR_PEG vs. CAR_CK (**A**) and the C22_PEG vs. C22_CK (**B**) comparisons. Red dots represent the upregulated genes with statistical significance, the blue dots represent the downregulated genes with statistical significance, and the grey dots (ns) are DEGs with −log10padj < 1.3, adopting a log_2_ Fold Change threshold of 1 (2.0 fold change). The X-axis is the gene expression change, and the Y-axis is the *p*-value adjusted after normalisation.

The characterisation of the citrus plant’s transcriptional response to drought stress was carried out by the identification of the unigenes whose expression level changed upon PEG treatment within each genotype. A total of 6092 DEGs (2119 upregulated and 3973 downregulated genes) were identified in the CAR_PEG vs. CAR_CK comparison (Figure 2A), whereas 320 DEGs (15 upregulated and 305 downregulated genes) were identified in the C22_PEG vs. C22_CK comparison, thus indicating that a limited PEG-induced transcriptomic reprogramming occurred in C22 and that nearly all genes were downregulated under drought conditions (Figure 2B). The validation of expression levels for nine selected DEG candidates was carried out by quantitative real-time PCR (qRT-PCR). The results show high congruence between RNA-Seq results and qRT-PCR analysis (coefficient of determination R^2^ = 0.99), indicating the reliability of RNA-Seq in the quantification of gene expression (Figure S3).

### 3.4. Functional Classification of DEGs

**Figure 3:**
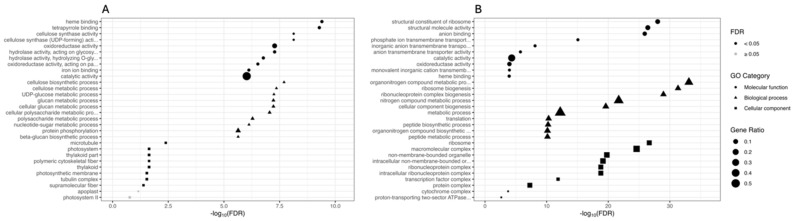
Gene Ontology (GO) enrichment analysis for the DEGs in the CAR_PEG vs. CAR_CK (**A**) and the C22_PEG vs. C22_CK (**B**) comparisons. The X-axis indicates the -log10(FDR), and the Y-axis indicates the GO terms within each category. Black dots indicate significantly enriched terms (FDR < 0.05), while grey dots indicate non-significantly enriched terms (FDR ≥ 0.05). Symbols indicate the GO category (circles indicate the Molecular function category, triangles indicate the Biological process category, and squares indicate the Cellular component category). The dot size indicates the Gene Ratio.

GO terms and KEGG pathway functional enrichments were performed to identify biological processes or pathways involved in drought stress response. We performed GO enrichment analysis on the set of DEGs comparing PEG-treated and CK plants within each genotype (CAR_PEG vs. CAR_CK and C22_PEG vs. C22_CK). Figure 3 shows the ten most significantly enriched terms within each GO category for each comparison. Considering the “Molecular function” category, “heme binding”, “tetrapyrrole binding”, and “cellulose synthase activity” are the three most enriched GO terms in the CAR_PEG vs. CAR_CK comparison (Figure 3A), while “structural constituent of ribosome”, “structural molecule activity”, and “anion binding” are the three most enriched GO terms in the C22_PEG vs. C22_CK comparison (Figure 3B). Among the ten most significantly enriched terms, both genotypes share only two significantly enriched terms: “oxidoreductase activity” (GO: 0016491) and “catalytic activity” (GO: 0003824). In the “Biological processes” category, “cellulose biosynthetic process”, “cellulose metabolic process”, and “UDP-glucose metabolic process” are the three most enriched GO terms in the CAR_PEG vs. CAR_CK comparison (Figure 3A). “Organonitrogen compound metabolic process”, “ribosome biogenesis”, and “ribonucleoprotein complex biogenesis” are the three most enriched GO terms in the C22_PEG vs. C22_CK comparison (Figure 3B). Finally, in the “Cellular component” category, “microtubule”, “photosystem”, and “thylakoid part” are the three most enriched GO terms in the CAR_PEG vs. CAR_CK comparison (Figure 3A), while “ribosome”, “macromolecular complex”, and “non-membrane-bounded organelle” are the three most enriched GO terms in the C22_PEG vs. C22_CK comparison (Figure 3B).

**Figure 4:**
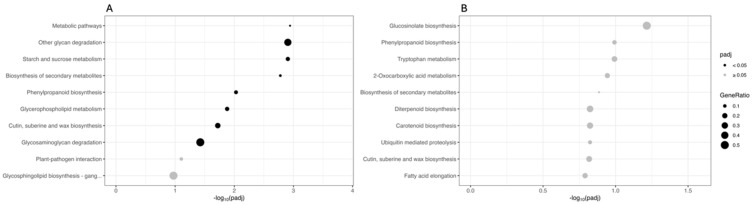
Kyoto Encyclopedia of Genes and Genomes (KEGG) enrichment analysis for the DEGs in the CAR_PEG vs. CAR_CK (**A**) and the C22_PEG vs. C22_CK (**B**) comparisons. The X-axis indicates the −log10(padj), and the Y-axis indicates the KEGG pathways. Black dots indicate significantly enriched terms (padj < 0.05), while grey dots indicate non-significantly enriched terms (padj ≥ 0.05). The dot size indicates the Gene Ratio.

The sets of DEGs originating from the two comparisons were also mapped onto the KEGG database. Figure 4 shows the main ten KEGG pathways, sorted by decreasing significance value (adjusted *p*-value). In the CAR_PEG vs. CAR_CK comparison, the most significantly enriched terms are “Metabolic pathways”, “Other glycan degradation”, and “Starch and sucrose metabolism”, indicating that a deep reprogramming of these metabolisms under drought conditions occurred. Other important pathways such as “Biosynthesis of secondary metabolites”, “Phenylpropanoid biosynthesis”, “Glycerophospholipid metabolism”, and “Cutin, suberin and wax biosynthesis” were also found to be deregulated by drought (Figure 4A). In contrast, in the C22_PEG vs. C22_CK comparison, no terms are significantly enriched (padj ≥ 0.05). However, the most represented terms are “Glucosinolate biosynthesis”, “Phenylpropanoid biosynthesis”, and “Tryptophan metabolism” (Figure 4B).

### 3.5. Identification of Functional Genes Related to Drought Stress Tolerance

To unravel citrus responses to drought stress, we analysed the RNA-Seq datasets from the two comparisons, focusing on genes known to be related to water scarcity, from drought sensing and signalling to the main downstream metabolisms [45,46]. With regard to the C22_PEG vs. C22_CK comparison, the subsets of DEGs associated with the three most significant GO terms in each category were specifically examined. Notably, among these, several genes related to cell cycle and growth were identified, including *C. clementina* protein PELPK1 and probable carboxylesterase 15, *C. sinensis* protein ENDOSPERM DEFECTIVE 1, *C. sinensis* G2/mitotic-specific cyclin S13-7, *C. sinensis* cell division control protein 6 homologue B, *C. clementina* microtubule-associated protein RP/EB family member 1C, *C. clementina* cell division cycle 20.2, and homologues to *A. thaliana* Cyclin-dependent kinase B2-2 and Protein POLLENLESS 3-LIKE 2 (Table 3). Interestingly, clusters specifically associated with the response to drought stress were observed in CAR_PEG vs. CAR_CK, which exhibited a more remarkable reorganisation of the transcriptome in response to water scarcity. Being worthy of attention, the following analysis was focused on the Carrizo genotype.

#### 3.5.1. Drought Sensing and Signalling

The analysis of differently expressed genes between CAR_PEG and CAR_CK samples revealed several Calmodulin-like proteins (CMLs), exhibiting both upregulation and downregulation in response to drought stress (Table 4). Specifically, *C. sinensis* calmodulin-like proteins 3 and 8, *C. sinensis* probable calcium-binding protein CML41, and homologue to *A. thaliana* Calmodulin-like protein 30 were found downregulated, whereas *C. sinensis* probable calcium-binding proteins CML11 and 16, *C. clementina* probable calcium-binding protein CML44, and homologue to *A. thaliana* Calcium-binding proteins CML37, 42, and 50 were found upregulated. CMLs act as calcium ion (Ca^2+^) sensors and serve as key components of calcium signalling networks in plants, playing pivotal roles in coordinating cellular responses to environmental stimuli and developmental cues [47]. In the comparison of CAR_PEG vs. CAR_CK, a cluster encoding the *Citrus clementina* 9-cis-epoxycarotenoid dioxygenase gene (NCED3) and a homologue to *A. thaliana* molybdenum cofactor sulfurase (ABA3), both encoding pivotal enzymes in ABA biosynthesis, exhibited increased expression in response to drought. Conversely, genes involved in ABA perception and signalling, such as those encoding homologues to *A. thaliana* PYR1-LIKE 7 (PYL7) and *A. thaliana* leucine-rich repeat (LRR) receptor-like kinase (RLK) (GUARD CELL HYDROGEN PEROXIDE-RESISTANT1, GHR1), playing a crucial role in the early ABA signalling cascade, showed downregulation. Nevertheless, a homologue to *A. thaliana* G-PROTEIN COUPLED RECEPTOR 2 (GCR2), encoding a plasma-membrane-localised ABA receptor known to initiate the ABA-induced responses, was upregulated. Several protein phosphatase 2C (PP2C) genes, including *SAG113* and *ABI1*, involved in ABA signalling, were upregulated under water stress conditions. Furthermore, an upregulated homologue of *Oryza sativa* Serine/threonine-protein kinase (SAPK9), which belongs to the SNF1-RELATED PROTEIN KINASE (SnRK2) family [48], was identified. This kinase plays a positive regulatory role in both ABA-responsive transcription factors and the downstream metabolic pathways influenced by ABA response. Finally, the *Responsive to Dehydration 29B* (*RD29B*) gene, a well-known ABA-induced stress-responsive gene, was induced by drought.

#### 3.5.2. ROS Scavenging

As shown in Table 4, clusters known to be involved in enzymatic ROS scavenging were retrieved. Our analysis revealed the upregulation of a cluster encoding *C. sinensis* Glutathione reductase (GR), a key player in ROS scavenging, involved in the reduction of glutathione disulfide (GSSG) to the sulfhydryl form glutathione (GSH) [49,50]. Additionally, the upregulation of a homologue *A. thaliana* Glutathione Peroxidase 1 (GPX1), which utilises glutathione to reduce H_2_O_2_ and is involved in the reduction of lipid and organic hydroperoxides [51], was observed. Moreover, a cluster encoding a homologue of *A. thaliana* L-ascorbate peroxidase S (APXS), which utilises ascorbic acid as its specific electron donor to reduce H_2_O_2_ to water [52], was upregulated in response to water scarcity in Carrizo (Table 4).

#### 3.5.3. Osmolyte Biosynthesis

A significant upregulation of *C. sinensis* 1-delta-pyrroline-5-carboxylate synthase (P5CS), a key enzyme in proline biosynthesis, was registered in the CAR_PEG vs. CAR_CK comparison (Table 4). Moreover, a homologue of *A. thaliana* ornithine-δ-aminotransferase (δ-OAT), involved in an alternative biosynthetic pathway of proline under stress conditions [53,54], was among the upregulated genes. The expression of a cluster encoding a homologue of *A. thaliana* choline monooxygenase (CMO) was induced by drought in the CAR_PEG vs. CAR_CK comparison. CMO catalyses the conversion of choline into betaine aldehyde, a crucial intermediate in the well-known osmoprotectant glycine betaine biosynthetic pathway [55]. The downregulation of a cluster encoding a homologue of *A. thaliana* Polyamine Oxidase 5 (PAO5), an enzyme responsible for the oxidation of polyamines resulting in H_2_O_2_ production, was also observed (Table 4). An upregulated cluster encoding for *C. clementina* Galactinol synthase 2 (GOLS2) was identified in the CAR_PEG vs. CAR_CK comparison (Table 4). Galactinol synthase plays a crucial role in the biosynthesis of raffinose family oligosaccharides (RFOs), which act as osmoprotectants in plants [56,57]. Two clusters encoding *C. sinensis* beta-amylase 1 (BAM1) and Alpha-amylase 3 (AMY3), both involved in carbohydrate metabolism, were found upregulated (Table 4). BAM1 acts specifically at the nonreducing ends of α-1,4–linked glucan chains producing β-maltose, and AMY3 collaborates with BAM1 in facilitating starch breakdown in the leaves during osmotic stress [58]. Finally, a cluster encoding a homologue of *A. thaliana* Branched-chain-amino-acid aminotransferase 2 (BCAT2), exhibiting upregulation, was identified (Table 4) in the CAR_PEG vs. CAR_CK comparison. BCAT2 is an enzyme that catalyses the transfer of an amino group from branched-chain amino acids (BCAAs), such as leucine, isoleucine, and valine, to α-ketoglutarate, forming branched-chain keto acids (BCKAs) and glutamate.

#### 3.5.4. Cell Wall Metabolism

The GO enrichment analysis of the CAR_PEG vs. CAR_CK comparison revealed a significant enrichment of terms associated with cell wall metabolism. Among them, we identified 33 DEGs implicated in cellulose synthesis, cell wall biogenesis, and modification (Table 5). Notably, our analysis revealed the presence of several cellulose synthase proteins, including homologues to *A. thaliana* Cellulose synthase-like protein B4 and Cellulose synthase-like protein G2, as well as homologues to *O. sativa* Cellulose synthase-like protein H2 and Cellulose synthase A catalytic subunit 4. Additionally, *C. sinensis* cellulose synthase-like protein D5, *C. sinensis* cellulose synthase A catalytic subunits 1, 2, and 4, and *C. clementina* cellulose synthase A catalytic subunits 7 and 8 were also identified. Several Xyloglucan endotransglucosylase/hydrolase (XTH) proteins, which play a crucial role in modifying the structure and properties of the cell wall in plants, were also identified including *C. sinensis* xyloglucan endotransglucosylase/hydrolase protein 2-like, 5, 9, 33, and B, as well as *C. clementina* probable xyloglucan endotransglucosylase/hydrolase protein 6. Finally, DEGs related to cell wall modification were also identified. These DEGs include *C. clementina* pectinesterase 1, *C. sinensis* pectinesterase 2, 8, and 53, *C. clementina* probable pectinesterase/pectinesterase inhibitor 25 and 61, *C. sinensis* probable pectinesterase/pectinesterase inhibitor 12 and 51, *A. thaliana* pectinesterase/pectinesterase inhibitor 6, 40, and 41, and *C. sinensis* polygalacturonase and *C. sinensis* pectate lyase-like. Notably, all of these DEGs exhibited downregulation in response to drought stress, except for those involved in hemicellulose biosynthesis (“Cellulose synthase-like protein B4, H2, and G2”), which showed upregulation.

### 3.6. Analysis of Transcription Factor Gene Families

**Figure 5:**
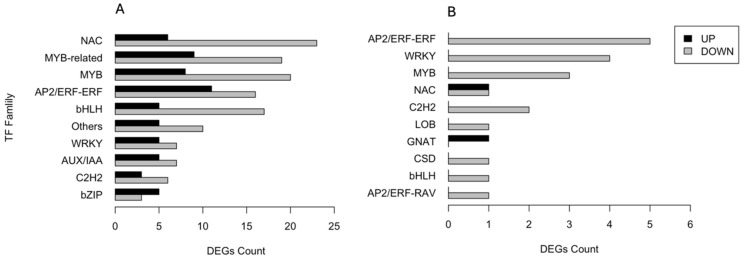
Distribution of the ten most abundant families of transcription factors responsive to drought stress in the CAR_PEG vs. CAR_CK (**A**) and the C22_PEG vs. C22_CK (**B**) comparisons.

A total of 345 DEGs encoding transcription factors (TFs) were identified and categorised into families. Figure 5 shows the number of both up- and downregulated DEGs across the ten most abundant families. In the CAR_PEG vs. CAR_CK comparison, 29 DEGs belong to NAC, 28 to MYB-related, 28 to MYB, and 27 to AP2/ERF-ERF families, respectively. The majority underwent downregulation in response to drought stress, except for the bZIP family (Figure 5A). Conversely, in the C22_PEG vs. C22_CK comparison (Figure 5B), only a total of 21 DEGs encoding TFs were recruited. All DEGs divided into ten TF families were downregulated by drought stress, except for those belonging to the NAC and GNAT families. In particular, five DEGs belong to AP2/ERF-ERF, four to WRKY, three to MYB, and two to NAC families, respectively (Figure 5B).

### 3.7. WCGNA Analysis

**Figure 6:**
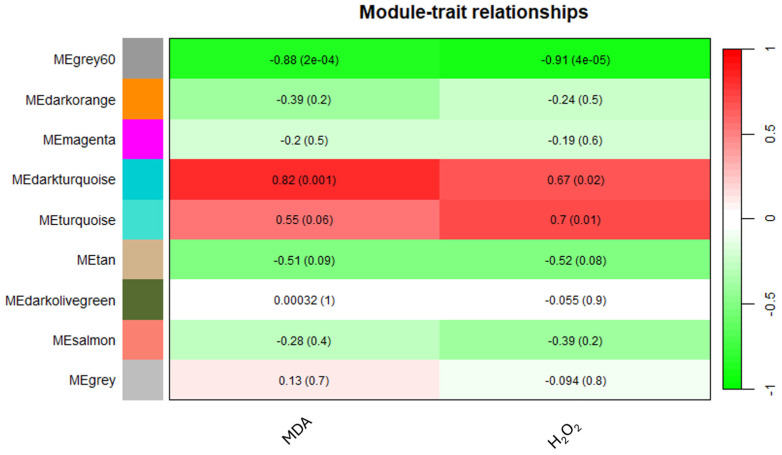
Heatmap of the correlation between modules and MDA and H_2_O_2_ levels.

The application of WGCNA based on the expression data of 6255 unigenes in orange leaves, considering an FPKM > 1 threshold, aimed to establish correlations between MDA or H_2_O_2_ levels and gene expression patterns. Using the parameters and thresholds indicated in the Materials and Methods section, these genes were grouped into nine co-expressed modules, each represented by a branch of the tree (Figure S4). The number of eigengenes in different modules is reported in Figure S5. The grey60 (1988), turquoise (1720) and darkorange (1220) modules include the highest number of eigengenes, whereas the grey60 module (13) gathered the lowest number of eigengenes (Figure S5). As shown in Figure 6, illustrating the module–trait relationships, the grey60 module exhibited significant negative correlations with both MDA and H_2_O_2_ contents, showing −0.88 and −0.91 correlation coefficients, respectively. Similarly, the darkturquoise module displayed significant positive correlations with MDA (0.82), whereas the turquoise module exhibited a significant correlation solely with H_2_O_2_ (0.7) (Figure 6).

**Figure 7:**
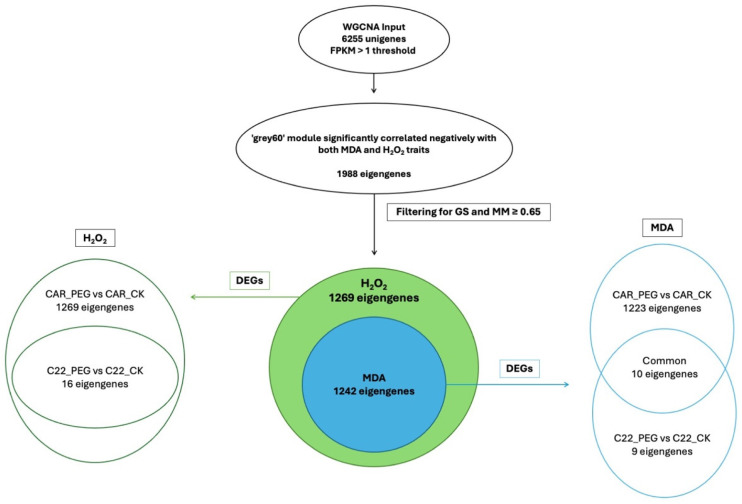
Diagrams illustrating the abundance and distribution of eigengenes associated with the grey60 module across various traits and comparisons.

**Figure 8:**
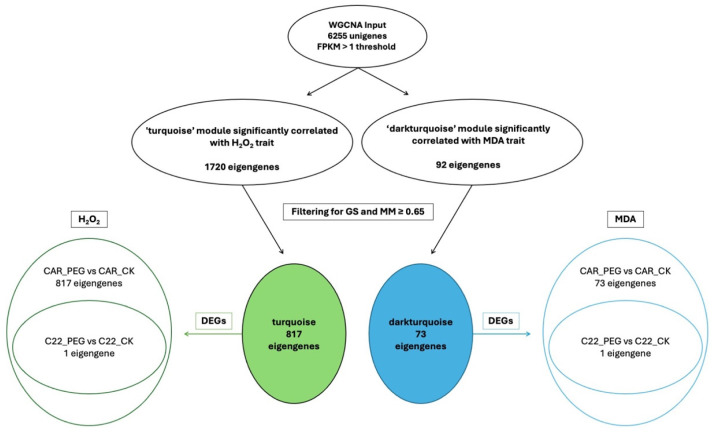
Diagrams illustrating the abundance and distribution of eigengenes associated with the turquoise and darkturquoise modules across various traits and comparisons.

In the WGCNA, GS indicates the correlation between a gene and a specific trait, while MM reflects the correlation between an individual gene and the module eigengene. Figure S6 illustrates the relationships between MM and GS for the traits of interest: MDA and H_2_O_2_. By filtering for MM and GS values ≥ 0.65, 1269 eigengenes correlated with H_2_O_2_ in the grey60 module and 1242 eigengenes were discovered to correlate with MDA. Intriguingly, all 1242 eigengenes correlating with MDA were also included within the 1269 eigengenes correlating with H_2_O_2_ (Figure 7), so that 27 genes were exclusively correlated with H_2_O_2_ content (Figure 7). Notably, all 1269 eigengenes correlating with H_2_O_2_ were DEGs in the CAR_PEG vs. CAR_CK comparison. Among them, only 16 genes were in the C22_PEG vs. C22_CK comparison (Figure 7). Similarly, a total of 1232 eigengenes correlating with MDA content were DEGs, and 1223 were within the CAR_PEG vs. CAR_CK comparison, whereas only nine genes were DEGs exclusively in the C22_PEG vs. C22_CK comparison. Finally, only 10 genes were in common between the comparisons (Figure 7). Within the turquoise module positively correlating with H_2_O_2_ content, all 817 eigengenes were DEGs in the CAR_PEG vs. CAR_CK comparison, whereas only one eigengene was differentially expressed in the C22_PEG vs. C22_CK comparison (Figure 8). Similarly, in the darkturquoise module, positively correlating with MDA levels, a total of 73 eigengenes were discovered, all of them being DEGs in CAR_PEG vs. CAR_CK comparison, whereas only one eigengene was differentially expressed in the C22_PEG vs. C22_CK comparison (Figure 8).

#### Transcription Factors Among the Eigengenes in Each Module

The genes belonging to the aforementioned modules underwent filtering based on their classification as TFs. Among them, several TFs are associated with the regulation of secondary wall biosynthesis, plant growth, and drought response in the CAR-PEG vs. CAR_CK comparison (Table 6). With regard to cell wall biogenesis, five TFs were identified within the grey60 module. These include genes coding a homologue of *C. clementina* NAC domain-containing protein 104 (NAC104/XND1), a homologue of *C. clementina* transcription repressor OFP4 (OFP4), and a homologue of *A. thaliana* NAC domain-containing protein 37 (NAC037/VND1), which negatively correlate with both MDA and H_2_O_2_ levels. Conversely, *C. sinensis* NAC domain-containing protein 12-like (NAC012/SND1) and *C. sinensis* transcription factor MYB83 (MYB83) were found to negatively correlate exclusively with MDA content (Table 6). Genes coding proteins involved in growth dynamics showing a negative correlation with both MDA and H_2_O_2_ levels were also found in the grey60 module, specifically, a homologue of *C. clementina* Growth-regulating factor 7 (GRF7) and *C. sinensis* B-box zinc finger protein 20 (BBX20/BZS1). Moreover, genes coding proteins involved in growth dynamics also show a positive correlation with H_2_O_2_ levels within the turquoise module. In particular, a homologue of *A. thaliana* Homeobox-leucine zipper protein ATHB-12-like (ATHB-12), a transcription factor required for ABA-mediated growth inhibition but not for stomatal closure, and a *C. sinensis* probable N-acetyltransferase HLS1 (HLS1) were found upregulated within the turquoise module (Table 6). Remarkably, HLS1 is the sole gene among the selected TFs displaying deregulation in both genotypes (data not shown).

Finally, several genes coding TFs involved in response to drought showed correlation with either MDA or H_2_O_2_ contents in the grey60, turquoise, and darkturquoise modules. Within the grey60 module, a gene encoding *C. sinensis* homeobox-leucine zipper (HAT22/ABIG1) negatively correlates with both H_2_O_2_ and MDA amount. Instead, four TFs positively correlate with H_2_O_2_ within the turquoise module, namely, *C. sinensis* ethylene-responsive transcription factor RAP2-1-like (RAP2.1), a negative regulator of DREB-type activators, *C. sinensis* cell differentiation protein rcd1-like (RCD1), and two clusters encoding DREB-type genes, namely, *C. sinensis* dehydration-responsive element-binding protein 2A (DREB2A) and a homologue of *C. clementina* dehydration-responsive element-binding protein 3 (DREB3/TINY2) (Table 6). These genes act as transcriptional activators, crucial in regulating gene expression under drought-stress conditions. Finally, a gene coding *C. sinensis* transcription factor (MYB2), known to act as a positive regulator conferring tolerance to salt, cold, and dehydration stress in rice, was found to be positively correlated to MDA in the darkturquoise module (Table 6).

## 4. Discussion

The impact of abiotic and biotic stresses on plant and crop growth, as well as on reproduction, is substantial. Unravelling the intricate molecular mechanisms of the cellular responses to these stresses is crucial for addressing challenges related to climate change and food security. Recent studies have investigated the transcriptome changes of various citrus rootstock genotypes under drought stress by RNA-Seq. Sweet orange [*Citrus sinensis* (L.) Osbeck var. Westin] grafted onto Rangpur lime (*Citrus limonia* Osbeck, “Santa Cruz” selection) or “Flying Dragon” trifoliate orange [*Poncirus trifoliata* (L.) Raf.] were exposed to a progressive soil water deficit by reducing the irrigation volume [59]. In that study, drought tolerance was shown to be related to the activation of genes involved in cell wall metabolism, soluble carbohydrates, and antioxidants, along with the downregulation of genes associated with starch metabolism, light reactions, and the ethylene signalling pathway [59]. More recently, the transcriptome analysis of “Longhuihong” navel orange (*Citrus sinensis* Osbeck cv. Longhuihong), a bud mutant of “Newhall” navel orange, along with “Newhall” navel orange (*Citrus sinensis* Osbeck cv. Newhall), both grafted on trifoliate orange (*Poncirus trifoliata* (L.) Raf.) and exposed to drought stress, suggested that the heightened tolerance of the mutant is associated with elevated levels of total waxes and aliphatic wax compounds, proline, soluble sugar, and significantly enhanced ROS-scavenging activities [60].

In this work, the results of RNA sequencing and the de novo assembly of two *Citrus* rootstock genotypes under PEG-induced drought stress are reported. The phenotype (Figure S1) and MDA levels clearly indicated a minimal impact of drought on Bitters plants. In this genotype, MDA levels slightly increased under drought, but they were not significantly higher than those in control plants. Differential gene expression analysis revealed a very low number of DEGs between C22 PEG-treated and control plants, mostly downregulated, suggesting that only a very moderate rearrangement of the transcriptome in response to stressful conditions occurred, with no identification of genes known to be directly involved in the response to drought stress. However, the observed downregulation of genes mainly related to the cell cycle and plant growth suggests that C22 plants modulated growth rates to adapt to challenging conditions under PEG treatment. On the contrary, Carrizo Citrange plants exhibited marked susceptibility to water stress, as pointed out by both phenotypic changes and the significant increase in MDA and H_2_O_2_ levels compared to control plants. Moreover, the upregulation of various ROS-scavenging enzymes observed in Carrizo plants under drought stress indicates the plant’s active response to mitigate the excessive accumulation of ROS. Similarly, the increased expression of genes involved in osmolyte biosynthesis (proline, glycine betaine, galactinol, and maltose) are evident indicators of stress-overcoming attempts to regulate osmotic balance. An essential physiological characteristic of drought-tolerant plants is their ability to increase cell wall elasticity and limit transpiration during drought conditions [61]. In Carrizo plants, a total of 27 DEGs implicated in cell wall biosynthesis and modification have been identified, the majority of which were downregulated under drought stress conditions. Among these DEGs, we found several genes encoding enzymes that play a critical role in modifying the structure and properties of the cell wall, aiding in cell wall loosening, a crucial process for plant adaptation to drought stress [62]. The involvement of these cell-wall-related proteins in plant response to drought stress has been documented in several previous studies and, in particular, Cellulose Synthase-Like D5 (CSLD5) has been proposed to be indispensable for osmotic stress tolerance in *Arabidopsis*, as mutants with decreased *CSLD5* expression accumulate elevated ROS levels under osmotic stress and exhibit heightened sensitivity to the oxidative stress [63]. The downregulation of these genes encoding cell-wall-related proteins in Carrizo plants under drought stress suggests their potential vulnerability to such conditions.

The development of resistance to drought stress in plants involves intricate physiological signalling pathways, including ABA (abscisic acid)- and ROS (reactive oxygen species)-induced pathways, as well as calcium ion (Ca^2+^) currents. The synthesis, sequestration, transportation, and turnover of ABA play a pivotal role in regulating critical abiotic stress responses, specifically influencing water balance and osmotic stress tolerance under conditions of drought and salt stress [64]. In our investigation, elevated expression levels of a gene homologous to *Citrus clementina* 9-cis-epoxycarotenoid dioxygenase (NCED3) and another homologous to *A. thaliana* molybdenum cofactor sulfurase (ABA3) in Carrizo were observed. These genes encode crucial enzymes involved in ABA biosynthesis whose heightened expression suggests that ABA synthesis persisted after 10 days under drought-stress conditions. Once synthesised, ABA initiates an elevation in Ca^2+^ currents by activating H_2_O_2_ production, a significant component of ROS in guard cells [65]. Calmodulin-like proteins, known as CMLs, emerge as potential candidates for Ca^2+^-binding proteins, acting as sensors to perceive and transmit the Ca^2+^ signal in cellular signalling pathways [47], including their involvement in responding to various abiotic stress factors such as drought, salt, and osmotic stress [66]. However, it is intriguing that certain *At*CMLs function as negative regulators of the ABA accumulation induced by drought stress. Therefore, our data suggest that the differentially expressed CMLs, whether functioning as positive or negative regulators, are implicated in the drought signalling pathway in Carrizo. ABA possesses various receptors, including ABAR/CHLH, GCR2, GTG1/2, and PYR/PYL/RCAR. Specifically, the PYR/PYL/RCAR protein attaches to ABA molecules outside the cell membrane. This interaction then hinders the phosphatase activity of the downstream protein phosphatase PP2C [67]. The deactivation of PP2C results in the accumulation of the active configuration of SNF1-RELATED PROTEIN KINASE (SnRK2) that, in turn, serves as a positive regulator for ABA-responsive transcription factors, as well as for downstream ABA-responsive metabolic pathways. Nevertheless, we registered the downregulation of two genes associated with early ABA signalling pathways. These genes are homologous to *A. thaliana* PYR1-LIKE 7 (PYL7) and *A. thaliana* leucine-rich repeat (LRR) receptor-like kinase (RLK), known as GUARD CELL HYDROGEN PEROXIDE-RESISTANT1 (GHR1). These findings indicate that PYL7 and GHR1 might not be active participants in the ABA signalling pathways in Carrizo plants under drought stress. In our analysis, several PP2C genes, including *SAG113* and *ABI1*, were upregulated under water stress conditions. PP2Cs are well known for their negative regulatory impact on ABA signalling [68]. During drought stress, modulating ABA responses can be crucial for both plant survival and growth, and it could involve adjusting the sensitivity of cells to ABA or regulating the downstream signalling pathways to promote growth under stress conditions.

Transcription factors stand as pivotal regulators, governing the expression of a broad range of target genes and consequently impacting the level of drought tolerance in plants. They play an important role in converting stress-induced signals to cellular responses. The WGCNA analysis enabled the identification of TFs whose expression showed positive or negative correlations with the levels of MDA and/or H_2_O_2_. Among them, a group of TFs within the grey60 module exhibiting significant negative correlations with MDA content are related to cell wall biosynthesis and are all downregulated in Carrizo plants. In particular, NAC012/SND1 involves the binding to the promoter regions of *Myb46*, thus leading to lignin biosynthesis induction.

*MYB83* is known to be directly activated by NAC012/SND1 displaying a coherent expression pattern [69]. MYB83 facilitates the activation of downstream transcription factors and a suite of genes crucial for secondary wall biosynthesis, cell wall modification, and programmed cell death pathways [70], representing an additional tier of master switches essential for regulating various aspects of secondary wall formation [69]. The downregulation observed in MYB83 aligns with NAC012/SND1 expression changes in Carrizo and accounts for the impairment of cell wall biosynthesis in Carrizo plants under drought. Studies have proposed that NAC104/XND1 acts as a negative regulator of secondary cell wall fibre synthesis and programmed cell death in *Arabidopsis* [71]. OFP4 protein belongs to a plant-specific family of regulatory proteins functioning as transcriptional repressors, exerting a negative regulatory influence on secondary cell wall formation [72,73,74]. These findings underscore the redundancy and interplay among transcription factors governing secondary wall biosynthesis processes in plants. Moreover, since both positive regulators (such as NAC012/SND1 and MYB83) and negative regulators (NAC104/XND1 and OFP4) of secondary cell wall synthesis were discovered to be downregulated, tight control of secondary wall synthesis probably occurred in response to drought stress in Carrizo plants.

Two TFs related to plant growth were found within the grey60 module, i.e., GRF7 and BZS1, respectively known for their involvement in the regulation of cell expansion [75] and function as a positive regulator of seedling photomorphogenesis. On the contrary, *ATHB12* and *HLS1*, two TFs within the turquoise module, are upregulated and positively correlated with H_2_O_2_ content in Carrizo. ATHB12 is proposed to function as a negative regulator of growth and its induction is contingent upon the presence of ABA [76]. The HLS1 gene is implicated in negatively modulating both sugar and auxin signalling pathways in *Arabidopsis* [77]. Taken together, the regulation of their gene expression corroborates the global idea that drought reduces cell growth in Carrizo plants by orchestrating the expression of both positive and negative growth regulation. In particular, the upregulation of HLS1 in Carrizo plants might indicate that the suppression of auxin signalling through HLS1 is part of Carrizo’s response to drought stress. A group of six TFs whose function is related to drought response were discovered within the turquoise (4), grey60 (1), and darkturquoise (1) modules. The four TFs within the turquoise module negatively correlate with H_2_O_2_ content: RAP2.1, RCD1, DREB2A, and DREB3. RAP2.1 possesses an APETALA2 (AP2) domain binding to dehydration-responsive elements (DREs) and acts as a negative regulator of DREB-type activators. RAP2.1 undergoes robust induction in response to drought and cold stress via an ABA-independent pathway. *Arabidopsis* plants overexpressing RAP2.1 exhibit heightened sensitivity to cold and drought stresses [78], whereas the *Arabidopsis* mutant rcd1 displays reduced sensitivity to ABA, ethylene, and methyl jasmonate, leading to alterations in the expression of hormonally regulated genes and increased stomatal conductance compared to the wild-type [79]. RAP2.1 is upregulated in Carrizo under drought conditions, probably being part of an unfavourable gene list determining the sensitiveness to water deficit. Two DREB proteins, DREB2A and DREB3, were found upregulated in this work. DREB2A is recognised as a pivotal transcription factor involved in triggering gene expression during high-salt and dehydration stress in *Arabidopsis* vegetative tissues [80] as its overexpression confers significant drought stress tolerance [81]. The induction of DREB3 was observed following treatments with ABA, cold, drought, and wounding [82]. HAT22 negatively correlates with both MDA and H_2_O_2_ contents within the grey60 module and plays a crucial role in ABA-mediated growth inhibition during drought stress [83]. MYB2 is known to act as a positive regulator, conferring tolerance to salt, cold, and dehydration stress in rice. It has been demonstrated that plants overexpressing *Os*MYB2 displayed increased accumulation of compatible osmolytes such as soluble sugars, free proline, and LEA proteins, while concurrently reducing the accumulation of MDA and H_2_O_2_ [84]. Concordantly, the deregulation of these latter genes (DREB2A, DREB2B, HAT22, and MYB2), along with their function being evaluated in other species in conferring stress tolerance, suggests that they can be objects of further future work aimed to generate stress-tolerant citrus rootstocks using genome editing approaches.

## 5. Conclusions

In this study, we exposed two citrus rootstock genotypes, Carrizo Citrange and Bitters (C22), to PEG-induced drought stress in a hydroponic system to evaluate their transcriptomic responses. C22 plants exhibited a minimal response to 10-day drought stress, as evidenced by limited phenotypic impact, non-significant MDA level changes, and minimal transcriptomic reprogramming. On the other hand, Carrizo plants sharply responded to water scarcity, attempting to withstand the stressful conditions. A putative induction of the ABA-mediated responses to water deficit was registered, as indicated by the deregulation of key ABA biosynthetic genes and other genes implicated in ABA signalling. However, the upregulation of certain negative ABA regulators suggests a potential modulation of ABA-induced drought response to mitigate the negative impact of sustained ABA signalling on plant growth. WGCNA suggests that Carrizo plants reprogrammed their metabolic priorities, trying to shift from growth-related processes towards the activation of mechanisms tailored for drought response by deregulating specific TFs. The response of Carrizo to PEG-induced osmotic stress involves a robust engagement of osmolytes, evidenced by the upregulation of proline, glycine betaine, and RFO biosynthetic genes, alongside the downregulation of a polyamine catabolic gene. The significant modulation of gene expression related to cell wall metabolism represents a critical response to drought stress in Carrizo plants. The robust response of Carrizo plants to stress provides valuable insights into the strategies employed to cope with drought, offering potential candidate genes for enhancing drought tolerance in Citrus.

## Figures and Tables

**Table 1:** Summary statistics of the RNA quality and sequencing results.

Clean reads	387 million
N° of transcripts	190,539
N° of unigenes	55,679
Average of read mapping rate	77.22%
Transcripts N50 (bp)	2544
Unigenes N50 (bp)	2384
Q30 (%)	91.1
GC content (%)	44.0

**Table 2:** The number and percentage of successfully annotated genes.

Database	Number of Unigenes	Percentage %
Annotated in Nr	34,859	62.60
Annotated in Nt	36,131	64.89
Annotated in KO	13,180	23.67
Annotated in Swiss-Prot	26,600	47.77
Annotated in Pfam	24,824	44.58
Annotated in GO	24,822	44.58
Annotated in KOG	8939	16.05
Annotated in at least one database	43,905	78.85

**Table 3:** List of DEGs related to drought stress response identified in C22_PEG vs. C22_CK comparison.

Cluster ID	Database Description	e-Value	log_2_ Fold Change
Plant growth			
625.32674	*Citrus clementina* protein PELPK1 (LOC18034992) (Nt ID: XM_006423706)	0	−6.009
625.11069	*Citrus clementina* probable carboxylesterase 15 (LOC18038652) (Nt ID: XM_006429930)	0	−2.649
Regulation of the cell cycle			
625.33484	Cyclin-dependent kinase B2-2 OS = *Arabidopsis thaliana* (Swiss-Prot ID: Q8LG64)	4.50 × −10^5^	−2.765
14231.0	*Citrus sinensis* protein ENDOSPERM DEFECTIVE 1 (LOC102631413) (Nt ID: XM_006467192)	0	−2.174
625.2046	*Citrus clementina* microtubule-associated protein RP/EB family member 1C (LOC18035782) (Nt ID: XM_006425920)	0	−1.241
625.1752	*Citrus clementina* cell division cycle 20.2, cofactor of APC complex (LOC18038983) (Nt ID: XM_006429932)	0	−2.615
625.9002	*Citrus sinensis* G2/mitotic-specific cyclin S13-7 (LOC102607074) (Nt ID: XM_006481988)	0	−2.593
14148.0	*Citrus sinensis* cell division control protein 6 homologue B (LOC102615076) (Nt ID: XM_006488435)	0	−2.592
625.1221	Protein POLLENLESS 3-LIKE 2 OS = *Arabidopsis thaliana* (Swiss-Prot ID: Q9SD20)	8 × 10^−144^	−3.245

**Table 4:** List of DEGs related to drought stress response identified in CAR_PEG vs. CAR_CK comparison.

Cluster ID	Database Description	e-Value	log_2_ Fold Change
Drought sensory and signalling mechanisms			
625.10101	*Citrus sinensis* calmodulin-like protein 3 (LOC102621120) (Nt ID: XM_006478639)	1.6 × 10^−51^	−2.926
625.8083	Calmodulin-like protein 30 OS = *Arabidopsis thaliana* (Swiss-Prot ID: Q9ZQE6)	9.4 × 10^−39^	−1.752
625.1956	*Citrus sinensis* calmodulin-like protein 8 (LOC102612336) (Nt ID: XM_006471604)	9.10 × 10^−36^	−4.987
625.14344	Calcium-binding protein CML42 OS = *Arabidopsis thaliana* (Swiss-Prot ID: Q9SVG9)	1.30 × 10^−10^	1.112
625.8942	*Citrus sinensis* probable calcium-binding protein CML41 (LOC102625955) (Nt ID: XM_006466645)	2.50 × 10^−37^	−2.324
625.12224	Calcium-binding protein CML37 OS = *Arabidopsis thaliana* (Swiss-Prot ID: Q9FIH9)	4.70 × 10^−29^	2.322
625.9804	*Citrus clementina* probable calcium-binding protein CML44 (LOC18041223) (Nt ID: XM_006434238)	8.70 × 10^−30^	1.744
625.19719	Probable calcium-binding protein CML50 OS = *Arabidopsis thaliana* (Swiss-Prot ID: Q9FYE4)	2.90 × 10^−41^	1.143
625.10631	*Citrus sinensis* probable calcium-binding protein CML16 (LOC102613292) (Nt ID: XM_006484317)	5.70 × 10^−51^	1.555
625.13944	*Citrus sinensis* probable calcium-binding protein CML11 (LOC102613046) (Nt ID: XM_006471463)	3.20 × 10^−57^	2.492
Hormone regulation of drought stress response			
625.13199	*Citrus clementina* 9-cis-epoxycarotenoid dioxygenase NCED3, chloroplastic (LOC18046011) (Nt ID: XM_006442173)	0	1.815
625.14504	Molybdenum cofactor sulfurase ABA3 OS = *Arabidopsis thaliana* (Swiss-Prot ID: Q9C5X8)	1.90 × 10^−145^	1.182
625.11871	Abscisic acid receptor PYL7 OS = *Arabidopsis thaliana* (Swiss-Prot ID: Q1ECF1)	7.80 × 10^−7^	−1.602
625.18189	LRR receptor-like serine/threonine-protein kinase GHR1 OS = *Arabidopsis thaliana* (Swiss-Prot ID: C0LGQ9)	1.10 × 10^−160^	−1.562
625.17411	LanC-like protein GCR2 OS = *Arabidopsis thaliana* (Swiss-Prot ID: F4IEM5)	2.70 × 10^−45^	1.376
625.23350	Probable protein phosphatase 2C 78 OS = *Arabidopsis thaliana* (Swiss-Prot ID: Q9FIF5)	2.30 × 10^−51^	2.009
625.22859	Protein phosphatase 2C 56 OS = *Arabidopsis thaliana* (Swiss-Prot ID: P49597)	4.30 × 10^−70^	1.778
625.32346	Serine/threonine-protein kinase SAPK9 OS = *Oryza sativa* subsp. japonica (Swiss-Prot ID: Q75V57)	7.70 × 10^−25^	1.285
625.19272	Low-temperature-induced 65 (RD29B) kDa protein OS = *Arabidopsis thaliana* (Swiss-Prot ID: Q04980)	3.30 × 10^−41^	2.362
ROS scavenging regulatory mechanisms			
625.36805	Peroxidase 5 OS = *Vitis vinifera* (Swiss-Prot ID: A7QEU4)	9.10 × 10^−87^	8.742
625.9703	Peroxidase 53 OS = *Arabidopsis thaliana* (Swiss-Prot ID: Q42578)	4.40 × 10^−129^	−5.654
625.20989	*Citrus sinensis* glutathione reductase (GR), cytosolic (LOC102614323) (Nt ID: XM_006493645)	0	1.211
625.20638	Phospholipid hydroperoxide glutathione peroxidase 1 (GPX1), chloroplastic OS = *Arabidopsis thaliana* (Swiss-Prot ID: P52032)	5.50 × 10^−76^	1.334
625.15425	L-ascorbate peroxidase S (APXS), chloroplastic/mitochondrial OS = *Arabidopsis thaliana* (Swiss-Prot ID: Q42592)	2.60 × 10^−65^	1.478
Osmolyte biosynthesis			
625.10562	*Citrus sinensis* delta-1-pyrroline-5-carboxylate synthase (P5CS) (LOC102609124) (Nt ID: XM_006486357)	0	1.371
625.33171	Ornithine aminotransferase (δ-OAT), mitochondrial OS = *Arabidopsis thaliana* (Swiss-Prot ID: Q9FNK4)	6.10 × 10^−87^	1.229
625.18915	Choline monooxygenase (CMO), chloroplastic OS = *Arabidopsis thaliana* (Swiss-Prot ID: Q9SZR0)	3.00 × 10^−79^	1.533
625.16432	Probable polyamine oxidase 5 (PAO5) OS = *Arabidopsis thaliana* (Swiss-Prot ID: Q9SU79)	1.40 × 10^−166^	−1.758
625.17328	*Citrus clementina* galactinol synthase 2 (GOLS2) (LOC18040981) (Nt ID: XM_024185001)	0	2.660
625.13485	*Citrus sinensis* beta-amylase 1 (BAM1), chloroplastic (LOC102626673) (Nt ID: XM_006493931)	0	1.180
625.23269	*Citrus sinensis* alpha-amylase 3 (AMY3), chloroplastic (LOC102577968) (Nt ID: XM_006483166)	0	1.401
625.9765	*Citrus clementina* branched-chain-amino-acid aminotransferase 2 (BCAT2), chloroplastic (LOC18041601) (Nt ID: XM_006433621)	0	3.743

**Table 5:** List of DEGs related to cell wall identified in CAR_PEG vs. CAR_CK comparison.

Cluster ID	Database Description	e-Value	log_2_ Fold Change
Cellulose synthesis			
625.8818	Citrus sinensis cellulose synthase-like protein D5 (LOC102621871) (Nt ID: XM_006479966)	0	−6.083
625.23407	Citrus clementina cellulose synthase A catalytic subunit 7 (LOC18053866) (Nt ID: XM_006453518)	0	−5.161
625.22336	Citrus sinensis cellulose synthase A catalytic subunit 4 (LOC102619893) (Nt ID: XM_006479401)	0	−5.098
625.19567	Citrus clementina cellulose synthase A catalytic subunit 8 (LOC18053215) (Nt ID: XM_006449474)	0	−4.836
625.31361	Citrus sinensis cellulose synthase A catalytic subunit 2 (LOC102610101) (Nt ID: XM_006464470)	0	−2.581
625.20272	Citrus sinensis cellulose synthase A catalytic subunit 1 (LOC102614848) (Nt ID: XM_006483275)	0	−2.527
625.13360	Cellulose synthase-like protein B4 OS = Arabidopsis thaliana (Swiss-Prot ID: O80891)	2.10 × 10^−32^	2.362
625.10234	Cellulose synthase-like protein H2 OS = Oryza sativa subsp. indica (Swiss-Prot ID: Q7PC71)	6.40 × 10^−9^	3.279
625.20767	Cellulose synthase A catalytic subunit 4 [UDP-forming] OS = Oryza sativa subsp. japonica (Swiss-Prot ID: Q5JN63)	6.50 × 10^−15^	3.449
625.28615	Cellulose synthase-like protein G2 OS = Arabidopsis thaliana (Swiss-Prot ID: Q8VYR4)	5.50 × 10^−16^	3.488
Cell wall biogenesis			
625.180	Citrus sinensis xyloglucan endotransglucosylase/hydrolase protein 2-like (LOC102609979) (Nt ID: XM_006479274)	0	−5.262
625.9604	Citrus sinensis probable xyloglucan endotransglucosylase/hydrolase protein 33 (LOC102608350) (Nt ID: XM_006483766)	0	−4.681
625.23478	Citrus clementina probable xyloglucan endotransglucosylase/hydrolase protein 6 (LOC18037502) (Nt ID: XM_006426099)	0	−4.521
625.934	Citrus sinensis probable xyloglucan endotransglucosylase/hydrolase protein B (LOC102617022) (Nt ID: XM_006487594)	0	−4.242
625.7212	Citrus sinensis xyloglucan endotransglucosylase/hydrolase 2-like (LOC102621846) (Nt ID: XM_006492934)	0	−2.966
625.9684	Citrus sinensis xyloglucan endotransglucosylase/hydrolase protein 9 (LOC102627322) (Nt ID: XM_006469681)	0	−2.958
625.33845	Citrus sinensis probable xyloglucan endotransglucosylase/hydrolase protein 5 (LOC102613907) (Nt ID: XM_006468883)	0	−2.386
Cell wall modification			
625.19798	Citrus sinensis pectate lyase-like (LOC102622327) (Nt ID: XM_006469872)	0	−6.881
625.13761	Pectinesterase/pectinesterase inhibitor 40 OS = Arabidopsis thaliana (Swiss-Prot ID: O81301)	4.80 × 10^−150^	−6.504
625.7348	Citrus clementina probable pectinesterase/pectinesterase inhibitor 25 (LOC18040524) (Nt ID: XM_006432471)	0	−6.256
625.24139	Pectinesterase 2 OS = Citrus sinensis (Swiss-Prot ID: O04887)	2.00 × 10^−70^	−5.624
14278.0	Probable pectinesterase/pectinesterase inhibitor 41 OS = Arabidopsis thaliana (Swiss-Prot ID: Q8RXK7)	6.70 × 10^−147^	−5.425
625.31477	Citrus sinensis pectinesterase 2-like (LOC102625023) (Nt ID: XM_006490034)	0	−5.013
625.30831	Citrus sinensis probable pectinesterase/pectinesterase inhibitor 12 (LOC102609650) (Nt ID: XM_006474494)	5.18 × 10^−156^	−4.460
625.38076	Citrus sinensis polygalacturonase-like (LOC102628485) (Nt ID: XM_006489884)	0	−3.731
625.24242	Citrus clementina probable pectinesterase/pectinesterase inhibitor 61 (LOC18047568) (Nt ID: XM_006441017)	0	−4.546
625.23761	Probable pectinesterase/pectinesterase inhibitor 6 OS = Arabidopsis thaliana (Swiss-Prot ID: O49298)	5.90 × 10^−92^	−3.668
625.15524	Citrus sinensis probable pectinesterase 53 (LOC102614801) (Nt ID: XM_025093060)	0	−3.342
625.7470	Citrus sinensis probable pectinesterase 8 (LOC102626812) (Nt ID: XM_006481381)	0	−3.232
625.19411	Citrus clementina pectinesterase 1 (LOC18036209) (Nt ID: XM_006426737)	0	−3.219
625.31930	Citrus sinensis probable pectinesterase/pectinesterase inhibitor 51 (LOC102610718) (Nt ID: XM_006464560)	0	−3.171
625.24083	Citrus sinensis probable polygalacturonase (LOC102617191) (Nt ID: XM_006476443)	0	−2.898
625.4677	Citrus sinensis probable polygalacturonase (LOC102629244) (Nt ID: XM_006490571)	0	−2.751

**Table 6:** List of DE TFs identified by the WGCNA analysis in the CAR_PEG vs. CAR_CK comparison.

Cluster ID	Database Description	e-Value	log_2_ Fold Change	Module_Traits
Cell wall				
13435.0	Citrus clementina NAC domain-containing protein 104 (NAC104/XND1) (LOC18042831) (Nt ID: XM_006432394)	0	−5.912	Grey60_MDA + H_2_O_2_
625.764	Citrus clementina transcription repressor OFP4 (LOC18041823) (Nt ID: XM_006431937)	0	−3.344	Grey60_MDA + H_2_O_2_
625.10943	NAC domain-containing protein 37 (NAC037/VND1) OS = Arabidopsis thaliana (Swiss-Prot ID: Q9SL41)	3.0 × 10^−113^	−2.45	Grey60_MDA + H_2_O_2_
625.8474	Citrus sinensis NAC domain-containing protein 12-like (NAC012/SND1) (LOC102629852) (Nt ID: XM_006476213)	0	−6.008	Grey60_MDA
625.613	Citrus sinensis transcription factor MYB83 (LOC102617737) (Nt ID: XM_006470693)	0	−2.206	Grey60_MDA
Growth				
625.24886	Citrus clementina growth-regulating factor 7 (GRF7) (LOC18049204) (Nt ID: XM_024188396)	0	−2.415	Grey60_MDA + H_2_O_2_
625.6370	Citrus sinensis B-box zinc finger protein 20 (BBX20/BZS1) (LOC102618175) (Nt ID: XM_006487266)	0	−2.081	Grey60_MDA + H_2_O_2_
625.19060	Citrus sinensis homeobox-leucine zipper protein ATHB-12-like (LOC102628407) (Nt ID: XM_006493109)	0	2.777	Turquoise_H_2_O_2_
625.28164	Citrus sinensis probable N-acetyltransferase HLS1 (LOC102617166) (Nt ID: XM_006493294)	0	3.431	Turquoise_H_2_O_2_
Drought response				
625.8123	Homeobox-leucine zipper protein HAT22 OS = Arabidopsis thaliana (Swiss-Prot ID: P46604)	1.4 × 10^−17^	−3.490	Grey60_MDA + H_2_O_2_
625.9832	Citrus sinensis ethylene-responsive transcription factor RAP2-1-like (LOC102627433) (Nt ID: XM_006473228)	0	2.231	Turquoise_H_2_O_2_
1379.0	Citrus sinensis cell differentiation protein rcd1-like (RCD1) (LOC102610349) (Nt ID: XM_015527477)	0	7.437	Turquoise_H_2_O_2_
625.14211	Citrus sinensis dehydration-responsive element-binding protein 2A (DREB2A) (LOC102610198) (Nt ID: XM_006486183)	0	1.371	Turquoise_H_2_O_2_
625.9371	Citrus clementina dehydration-responsive element-binding protein 3 (DREB3/TINY2) (LOC18055009) (Nt ID: XM_006450624)	0	2.904	Turquoise_H_2_O_2_
625.3658	Citrus sinensis transcription factor MYB2 (LOC102621399) (Nt ID: XM_006480200)	0	2.659	Darkturquoise_MDA

## Data Availability

The data presented in this study are openly available from the NCBI (https://www.ncbi.nlm.nih.gov/geo/ accessed on 5 August 2024, reference number PRJNA1126089).

## Supplementary Materials

The following supporting information can be downloaded at https://www.mdpi.com/article/10.3390/biology13080595/s1: Figure S1. Plant phenotypes. Figure S2. Transcript and unigene length distributions. Figure S3. RNA-Seq validation. Figure S4. Clustering dendrogram. Figure S5. Number of eigengenes. Figure S6. Gene significance and module membership. Table S1. Primer sequences.
