# Supplementary figures and images for: Transcriptome Profiling and Weighted Gene Correlation Network Analysis Reveal Hub Genes and Pathways Involved in the Response to Polyethylene-Glycol-Induced Drought Stress of Two Citrus Rootstocks
Source: Biology (Basel). 2024 Aug 7;13(8):595. doi: 10.3390/biology13080595 (PMC11351357; doi:10.3390/biology13080595)

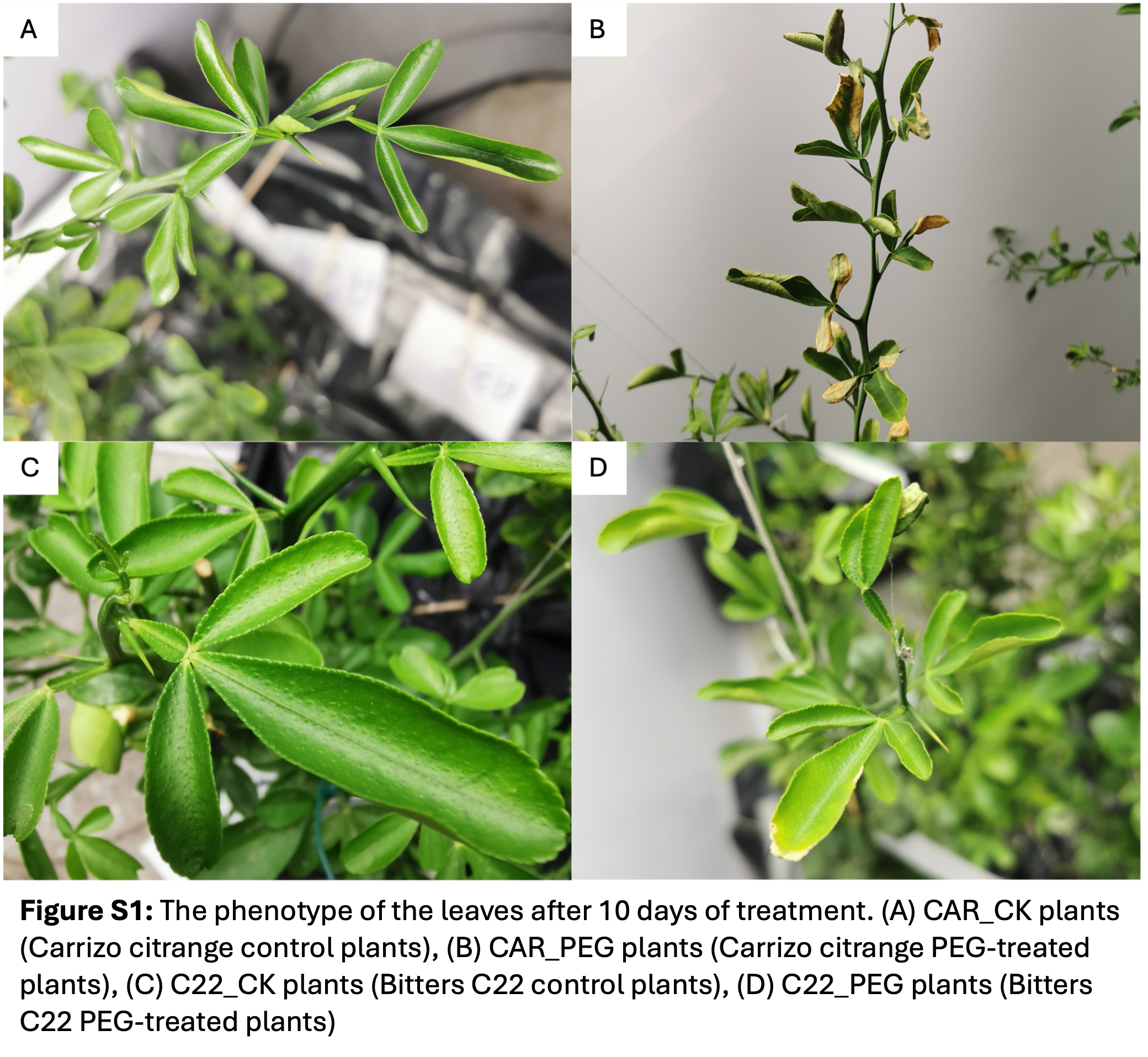

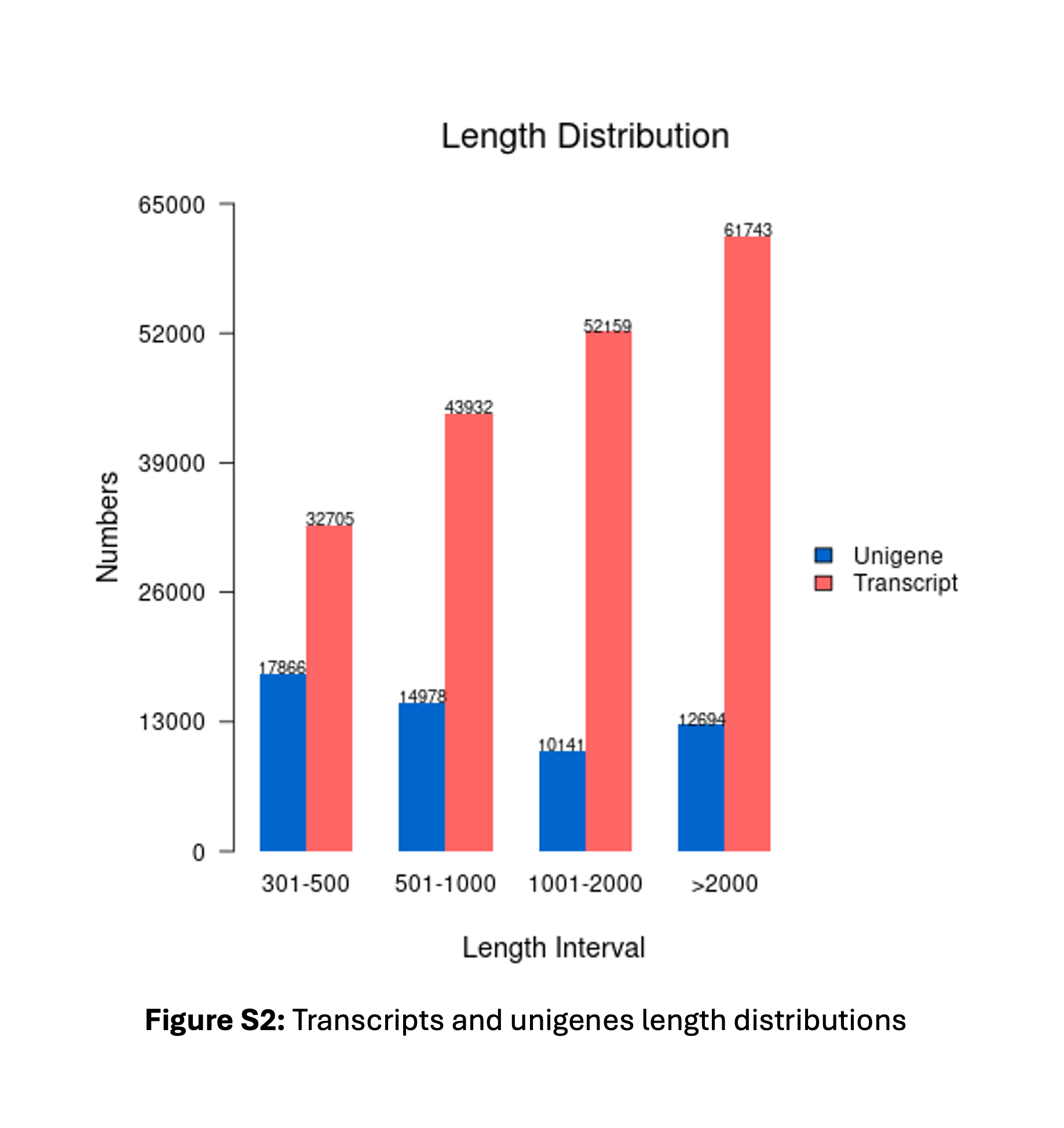

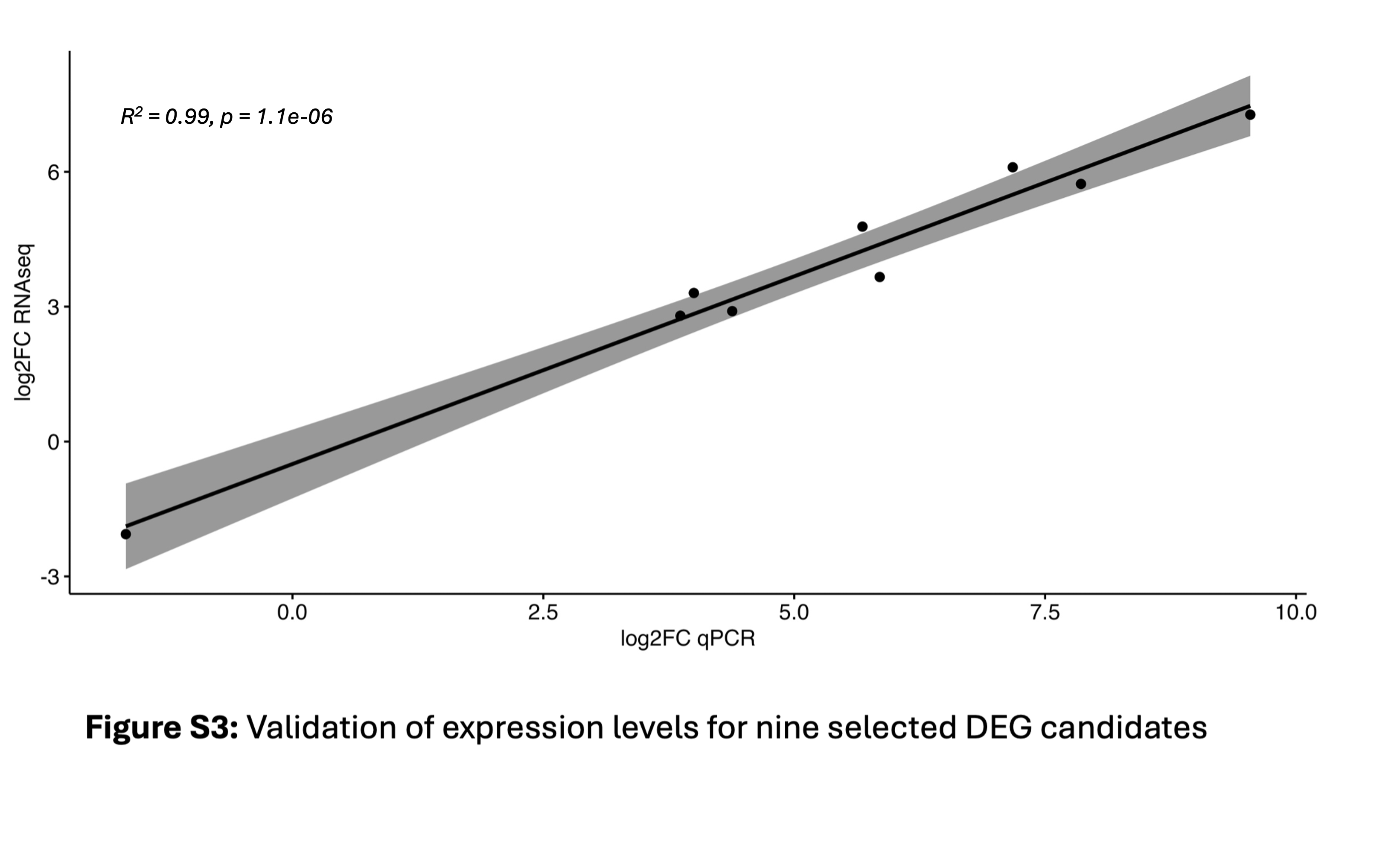

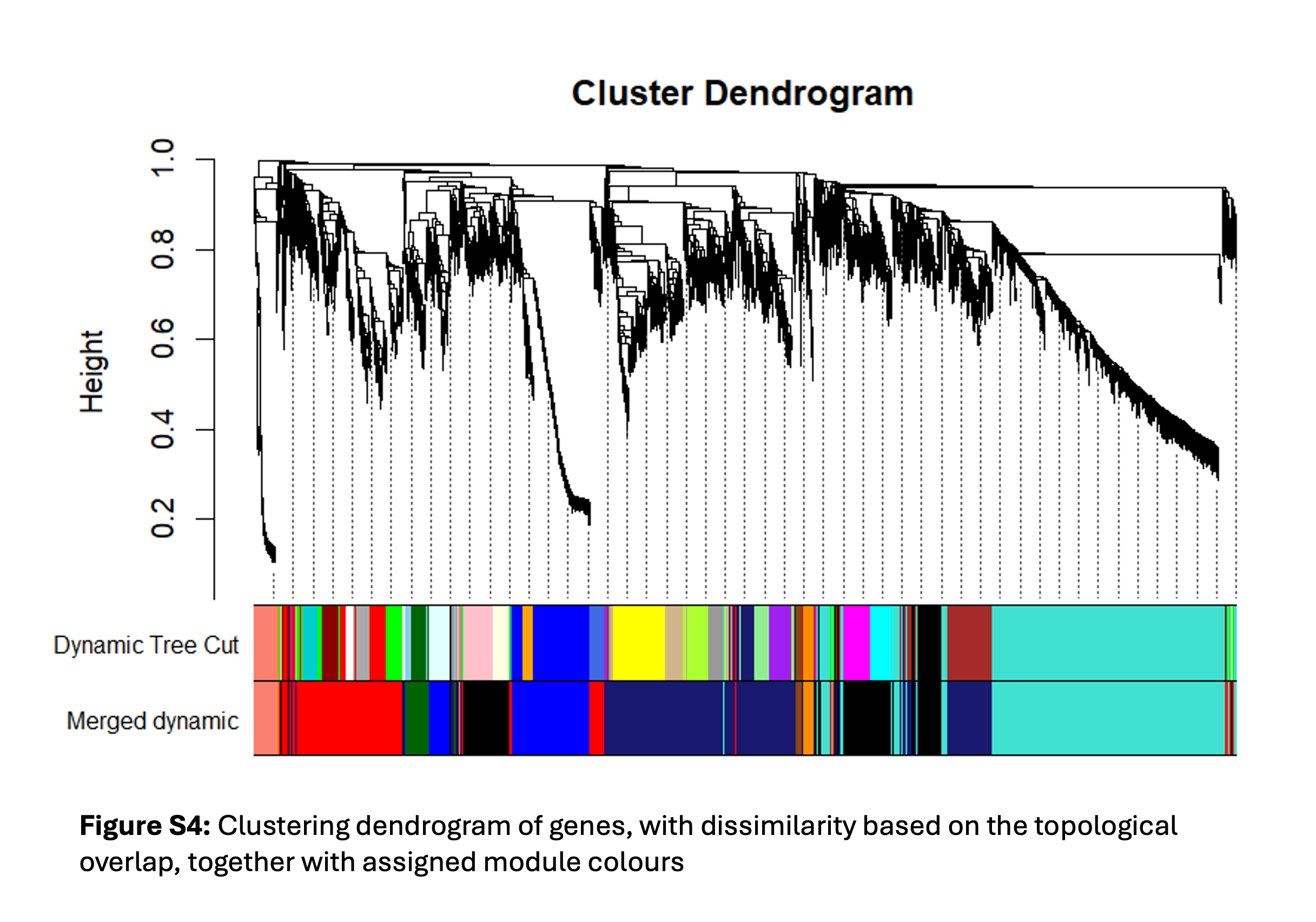

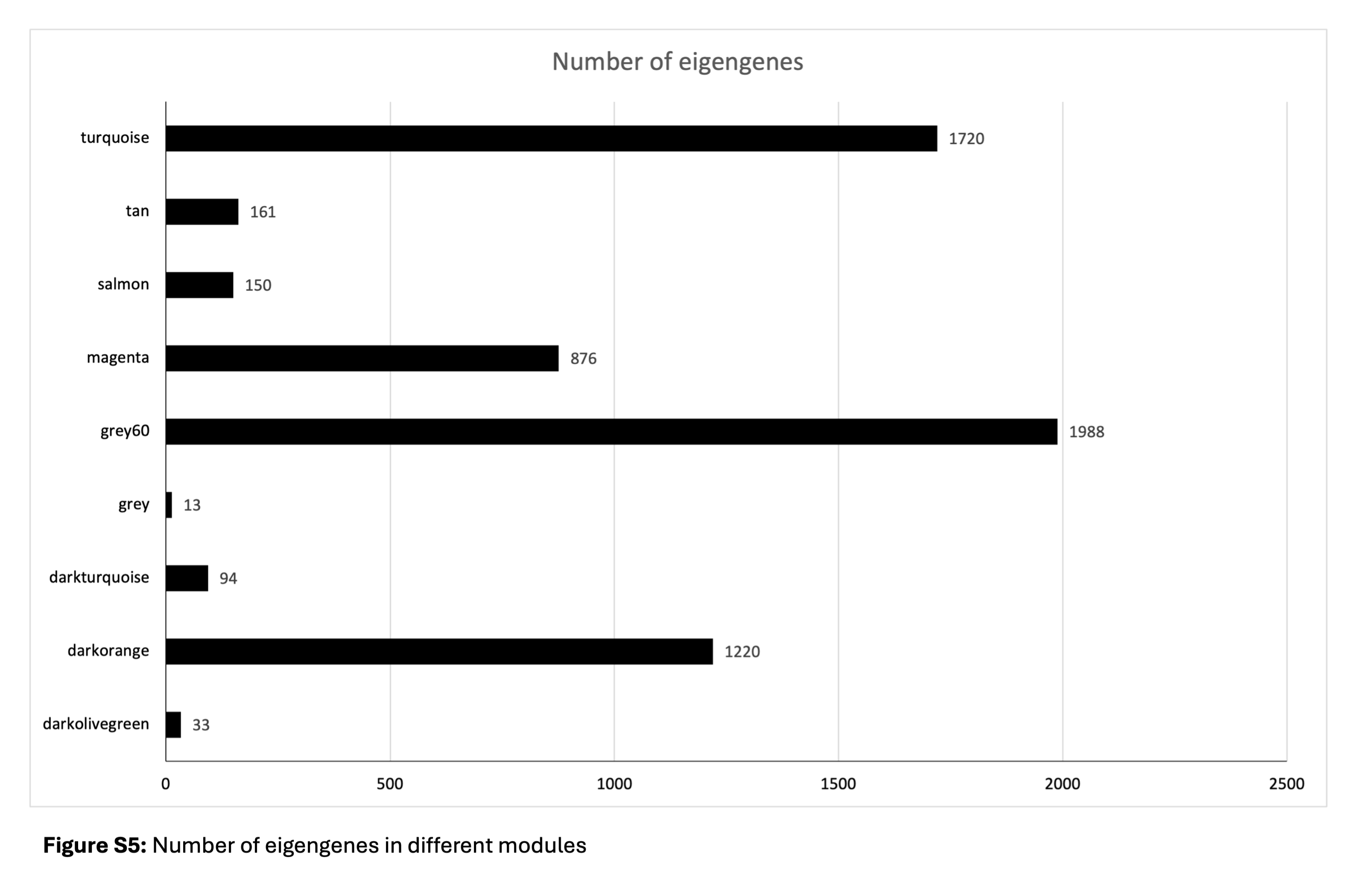

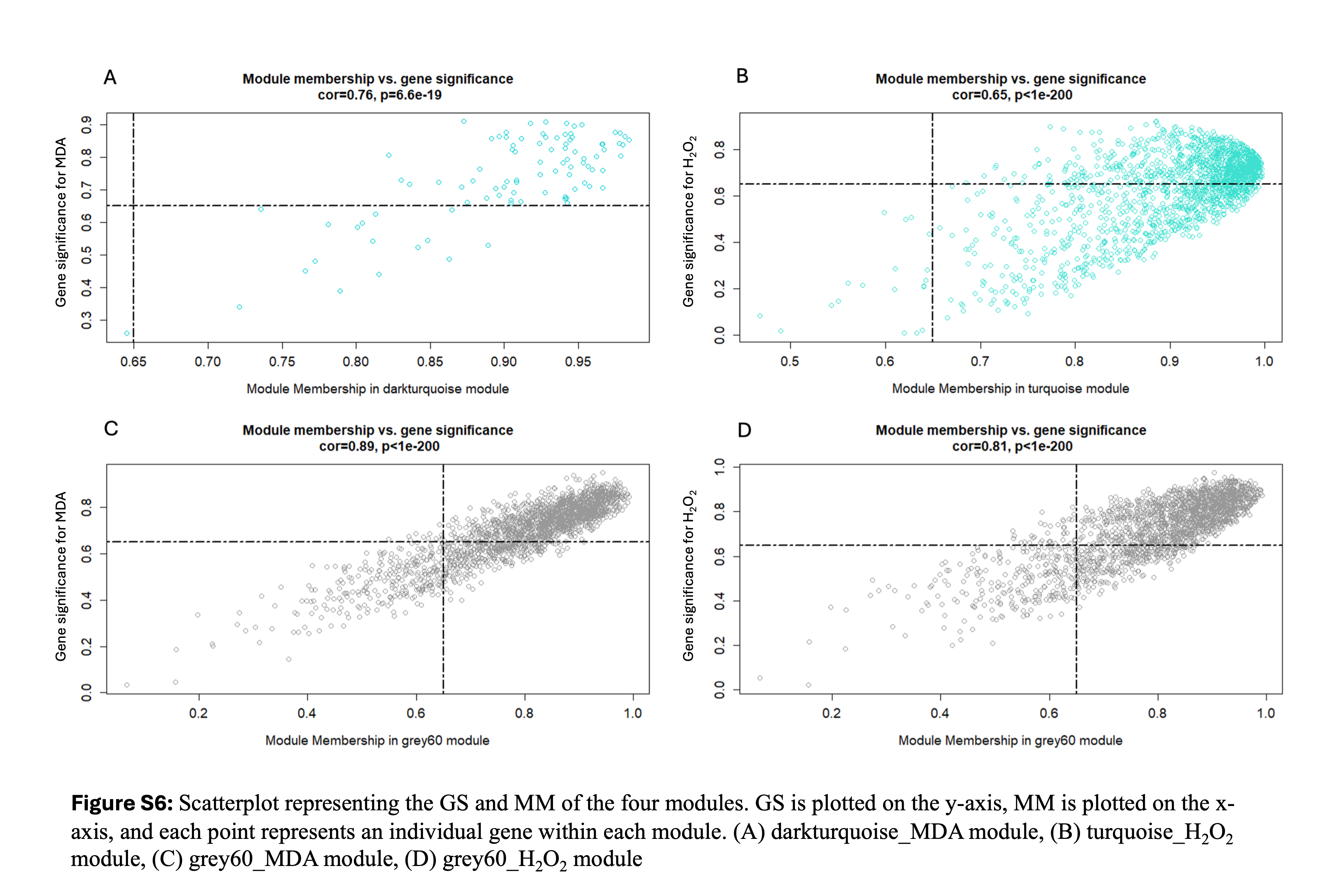

Supplement: Supplementary file 1 [file biology-13-00595-s001.zip › Figures.docx]
